# Evaluation of night eating syndrome and food addiction in esports players

**DOI:** 10.1007/s00394-024-03368-0

**Published:** 2024-03-23

**Authors:** Sedat Arslan, Ramazan Mert Atan, Nursel Sahin, Yasemin Ergul

**Affiliations:** Department of Nutrition and Dietetics, Bandirma Onyedi Eylul University, Balikesir, 10200 Turkey

**Keywords:** Esport, Esports players, Night eating syndrome, Food addiction, Health

## Abstract

**Purpose:**

Esports players’ training takes long periods and they sit for a long time during competitions, which increases their risk of obesity and urges them to develop inappropriate eating behaviors. In this study, we aimed to investigate the night-eating syndrome and food addiction in esports players.

**Methods:**

This cross-sectional study was conducted with 248 esports players who were members of a university’s esports community. The study data were collected using an online questionnaire consisting of the descriptive information form, Night Eating Questionnaire, and Yale Food Addiction Scale.

**Results:**

The mean age of the sports players participating in the study was 22.19 ± 5.97 years. Of them, 55.6% had a normal body weight, 13.4% were obese, 54.4% played esports for 3 years or more, 13.3% experienced night eating syndrome, and 21.4% experienced food addiction. While the weekly duration of playing esports and skipping meals were associated with night eating syndrome, the weekly duration of playing esports and smoking were associated with food addiction (*p* < 0.05). Additionally, although there was no statistically significant difference, the risk of food addiction was 2.12 times higher in those with poor perceived sleep quality.

**Conclusion:**

We observed that night eating syndrome was very common in esports players and that these individuals were at risk in terms of food addiction. Since esports has a more sedentary structure than traditional sports, we suggest that esports players should be evaluated in terms of their unhealthy eating behaviors and risk of eating disorders.

## Introduction

Esports, short for electronic sports, is a rapidly growing sport involving players competing in video game tournaments. Although electronic sports tournaments have origins dating back to the 1970s, organizing such tournaments professionally started in 1997 [1]. This discipline is becoming more and more popular among players and viewers. Esports are considered to have features similar to those of traditional sports, mainly both due to their competitive nature and due to the “physicality” and “skill” required to play esports at a high level [2].

Traditionally, sports nutrition has focused on promoting an optimum level of physical fitness, especially for sports people who do physically more intense sports such as marathons, football, and weightlifting. However, sports are not limited to these branches. Besides physically intense sports, there are also sports and competitive activities such as billiards, darts, chess, and esports that have high cognitive capacity, high-speed response, accuracy, and resistance to mental fatigue [3]. Various authorities predict that the number of esports viewers will increase from 435.9 million in 2020 to 577.2 million by 2024 [4]. Esports and esports players have become the focus of attention due to the increasing number of viewers and the economic ecosystem [5]. In line with this popularity, performance, and approaches that can increase performance have gained importance in esports players who participate in tournaments, take part in professional teams, and earn money from esports [6].

Esports competitions are stressful, esports players undergo long-term training and their exposure to fierce competition can adversely affect the health of the players [7, 8]. A recent systematic review and meta-analysis noted that some of the biopsychosocial risks for esports players include poor nutrition, caffeine supplementation, physiological arousal, pain, stress, cognitive fatigue, gaming addiction, and mental health problems [9, 10]. As a matter of fact, unlike traditional sports, it has been reported that excessive gaming at night reduces sleep time and increases the stress level of e-sports players [11]. Also, due to the nature of esports, esports players are supposed to sit for a long time during training and competition, which may increase the risk of obesity and encourage them to develop inappropriate eating behaviors [7]. In several experimental studies, it has been reported that excessive screen time and video games are associated with increased abdominal obesity [12, 13]. Abdominal obesity has also been associated with video games due to the increased risk of sedentary lifestyles, increased time spent on them, and insufficient physical activity [12]. It is known that esports players have obesity and sleep disorders due to long-lasting training and competitions [14, 15]. It has also been found that individuals with night eating syndrome (NES) and food addiction also have obesity and poorer sleep quality [16, 17]. . This situation suggests that esports players are at risk of NES and food addiction. The number of studies in which eating disorders in esports players are investigated is limited; therefore, in our study, we aimed to investigate NES and food addiction in esports players.

## Methods

### Study population

This cross-sectional study was conducted with esports players who were members of the esports community (*n* = 502) of a university located in the capital city of Turkey. The minimum sample size was calculated as 218 using the G*Power 3.1.9.7 program (power: 95%, α = 0.05, effect size d = 0.15). However, considering the possibility of losses during the study, we decided to include 15% (*n* = 30) more people (*n* = 248) [18, 19].

Between April 2022 and November 2022, individuals who were members of a university esports community and over the age of 18, and who agreed to participate in the study were included in the study.

### Study variables

While the dependent variables of the study were NES and food addiction, its independent variables were age, sex, marital status, Body Mass Index (BMI), esports level, weekly esports participation time, duration of being involved in esports, smoking, and alcohol use status, physical activity level, weekly sedentary time, number of meals per day, meal skipping, sleep duration, and perceived sleep quality.

### Data collection

We collected the study data using an online questionnaire consisting of the descriptive information form, Night Eating Questionnaire (NEQ), and Yale Food Addiction Scale (YFAS). The online survey was administered to the community members by the researchers via Discord (a social media platform where the members of the esports community come together, and chat via text, voice, or video in real-time with other people) application.

#### Descriptive information form

The form consists of 20 items questioning esports players’ age, sex, marital status, BMI, esports level, duration of esports training and competition, the duration they have been involved in esports, smoking, and alcohol use status, physical activity level, weekly sedentary time, the number of meals per day, meal skipping status, sleep duration and perceived sleep quality. The BMI of the participants was calculated based on their statements by dividing their weight (kg) by the square of their height (m^2^) (kg/m^2^) [20].

#### Night eating questionnaire - NEQ

The NEQ developed by Allison (2008) consists of 16 items whose responses are rated on a five-point Likert-type scale [21]. The minimum and maximum possible scores that can be obtained from the NEQ are 0 and 52, respectively. A score of 25 and above indicates the presence of NES. Turkish validity and reliability study of the NEQ was performed by Atasoy et al. (2014) and the Cronbach’s alpha coefficient was determined as 0.69 [22]. In our study, the value for Cronbach’s Alpha for the NEQ was α = 0.72.

#### Yale food addiction scale - YFAS

The 27-item YFAS (based on DSM-IV) developed to assess individuals’ food addiction status questions 7 criteria regarding the individuals’ eating habits in the last 12 months [23]. The YFAS was adapted to Turkish by Bayraktar et al. [24]. The validity and reliability study of the Turkish version of the YFAS was conducted by Sevinçer et al. (2014) and the Cronbach’s alpha value was determined as 0.859 [25]. In our study, the value for Cronbach’s Alpha for the YFAS was α = 0.74.

### Data analysis

The data were analyzed using the IBM SPSS Statistics for Windows. In descriptive statistics, the data were given as numbers, percentages, arithmetic mean, and standard deviation. In the comparison of mean scores obtained from the overall NEQ and YFAS and their sub-dimensions, Shapiro Wilk was used to confirm whether the data were normally distributed evaluated, and Student’s t, Mann Whitney U, ANOVA tests were used. In post hoc comparisons, the Kruskall Wallis H test was used if the data had normal distribution, and Bonferroni and Dunnet T3 tests were used if the variances were homogenous. Logistic regression analysis (enter method) was used to examine the relationship between the independent variables, night eating syndrome, and food addiction. Nagelkerke R2 statistics were used to evaluate the adequacy of the model, and parameters that could explain the variability in the dependent variable were included in the model. Age, sex, marital status, Body Mass Index (BMI), duration of being involved in esports, number of meals per day, sleep duration, and weekly sedentary time were used mainly for population descriptive purposes. Esports level, weekly esports participation time, smoking, and alcohol use status, physical activity level, number of meals per day, skipping meals, perceived sleep quality, and BMI were used for analysis of food addiction and some risk factors in the esports players. Along with these variables, sex also were used for analysis of NES and some risk factors in the esports players. The significance level of the statistical tests was accepted as *p* < 0.05.

### Ethical approval

Before the study was conducted, approval was obtained from the Bandirma Onyedi Eylul University Health Sciences Non-Interventional Research Ethics Committee (decision date: April 11, 2022, decision number: 2022-34). The study was conducted by the ethical principles of the Declaration of Helsinki.

## Results

The mean age of the sports players participating in the study was 22.19 ± 5.97 years. Of them, 84.3% were men, and only 11.7% were married. Their mean BMI was 24.61 ± 4.97 kg/m^2^. Of them, 55.6% were in the normal weight range and 13.4% were with obesity. While 60.1% of them were amateur esports players, 39.9% were professional esports players. Esports players’ 37.9% were smokers, 41.5% consumed alcohol, and 77.0% performed light sweating physical activities such as brisk walking, doing housework, carrying light loads, cycling at normal speed, or dancing which took them at least 150 min per week. As for their dietary habits, 69.8% of them skipped meals. Their mean weekly sleep duration was 51.98 ± 10.50 h, and 66.9% of them stated that their sleep quality was good (Table 1).

**Table 1:** Descriptive characteristics of the participants (*n* = 248)

Descriptive characteristics	*N* = 248	%
**Age** (Mean ± SD: 22.19 ± 5.97, Min: 18, Max:50 Median: 20) years		
**Sex**		
Women	39	15.7
Men	209	84.3
**Marital status**		
Single	219	88.3
Married	29	11.7
**BMI (**Mean ± SD: 24.61 ± 4.97, Min: 14.96, Max:47.32 Median: 23.7)) kg/m^2^		
**BMI classification** Underweight (< 18.5) Normal (18.5–24.9) Overweight (25–30) People with obesity (≥ 30)	15 138 62 33	6.0 55.6 25.0 13.4
**Esport Classification**		
Amateur Esports Player	149	60.1
Professional Esports Player	99	39.9
**Weekly Esports Playing Time (**Mean ± SD: 16.62 ± 16.66, Min: 1, Max: 90 Median: 10) hours/week		
**Durations the participants had been involved in esports (years)**		
0–1	52	21.0
1–3	61	24.6
> 3	135	54.4
**Smoking status**		
Non-smoker	151	60.9
At least one cigarette a day	75	30.2
Sometimes	19	7.7
Ex-smoker	3	1.2
**Alcohol consumption**		
No	145	58.5
Yes	103	41.5
**Doing physical activity at least 150 min per week**		
No	57	23.0
Yes	191	77.0
**Sedentary time** (Mean ± SD: 78.63 ± 22.17, Min: 50, Max: 161, Median: 71.5) hours		
**The number of the main meals consumed**		
1	24	9.7
2	114	46.0
3	97	39.1
≥ 4	13	5.2
**The number of snacks**		
1	117	47.2
2	81	32.7
3	25	10.1
≥ 4	12	4.8
None	13	5.2
**Skipping meals**		
Yes	173	69.8
No	75	30.2
**Meal skipped**		
Breakfast	104	41.9
Lunch	71	28.6
Dinner	10	4.0
Breakfast + Lunch	9	3.6
None	54	21.8
**Average Weekly Sleep Time (**Mean ± SD: 51.98 ± 10.50, Min: 21, Max: 108, Median: 50) hours		
**Perceived Sleep Quality**		
Good	166	66.9
Bad	82	33.1

Mean ± SD: Mean ± Standard Deviation. Min: Minimum, Max: Maximum

The distributions of the participants in terms of meeting the criteria for food addiction and their NES and food addiction status were given in Table 2. Of them, 21.4% experienced food addiction and 13.3% experienced NES. The mean score they obtained from the NEQ was 17.71 ± 6.21

**Table 2:** Participating esports players’ NES status and the mean score they obtained from the NEQ

YFAS Criteria		n	%
Substance taken in larger amount and for longer period than intended	Yes No	71 177	28.6 71.4
Persistent desire or repeated unsuccessful attempt to quit	Yes No	228 20	91.9 8.1
Much time/activity to obtain, use, recover	Yes No	84 164	33.9 66.1
Important social, occupational, or recreation activities given up or reduced	Yes No	78 170	31.5 68.5
Use continues despite knowledge of adverse consequences (e.g., failure to fulfill role obligation, use when physically hazardous).	Yes No	147 101	59.3 40.7
Tolerance (marked increase in amount; marked decrease in effect)	Yes No	209 39	84.3 15.7
Characteristic withdrawal symptoms; substance taken to relieve withdrawal	Yes No	52 196	21.0 79.0
Using leading to clinically significant impairment or distress	Yes No	53 195	21.4 78.6
**Food addiction**	No Yes	195 53	78.6 21.4
**NES**	No Yes	215 33	86.7 13.3
**NEQ Total score (Mean ± SD) min-max**	17.71 ± 6.21 Min: 4.0 Max: 40.0		

Mean ± SD: Mean ± Standard Deviation. Min: Minimum, Max: Maximum

As stated in Table 3, according to the results of our study, 54.5% of the participants with NES and 16.3% of those without NES experienced food addiction (*p* < 0.05)

**Table 3:** Evaluation of the relationship between NES and food addiction in the participants

	Those with NES		Those without NES		p
	n	%	n	%	
Those with food addiction	18	54.5	35	%16.3	0.000*
Those without food addiction	15	45.5	180	%83.7	
**TOTAL**	33	%100.0	215	%100.0	

*Pearson Chi-square, *p* < 0.05

The participating esports players’ risk factors for NES development were determined with the logistic regression analysis (Table 4). According to the results of the analysis, no relationship was determined between the NES risk and the variables such as sex, being an amateur/professional esports player, smoking, alcohol use, physical activity status, perceived sleep quality, and BMI (*p* > 0.05). The analysis of the parameters increasing the risk of NES demonstrated that a 1-hour increase in weekly esports duration and skipping meals increased the risk of NES development in the esports players 1.03 (OR: 1.034, *p* < 0.05) and 2.69 times (OR: 2.69, *p* < 0.05), respectively

**Table 4:** Univariate binary logistic regression analysis for NES versus no NES

Variable	Coef (B)	S.E.	Odds Ratio (Exp (B))	95 CI Upper Lower	p-value
**Sex** Women* Men	-0.851	0.764	0.427	0.096–1.909	0.265
**Esports level** Amateur esports players* Professional esports players	-0.671	0.452	0.511	0.211–1.240	0.138
**Weekly esports participation time (hours/week)**	0.036	0.017	1.034	1.002–1.067	**0.028**
**Smoking status** Non-smoker * Smoker	0.691	0.508	1.996	0.737–5.407	0.174
**Alcohol consumption** No* Yes	-0.441	0.481	0.643	0.250–1.652	0.359
**Physical activity**	-0.776	0.487	0.460	0.177–1.195	0.111
**The number of main meals** 1* 2 + 3	0.220 0.262	0.767 0.463	1.246 1.300	0.277–5.607 0.525–3.220	0.774 0.571
**Skipping meals** No* Yes	0.988	0.468	2.686	1.074–6.716	**0.035**
**Perceived sleep quality** Good* Bad	-0.129	0.482	0.879	0.342–2.262	0.789
**BMI** Underweight (< 18.5)* Normal (18.5–24.9) Overweight (25–30) People with obesity (≥ 30)	0.958 0.248 0.302	1.051 0.679 0.751	2.608 1.281 1.352	0.332–20.451 0.339–4.844 0.310–1.909	0.362 0.715 0.688

Logistic regression analysis, OR: odds ratio; CI: confidence interval

*Because the “reference” category for the categorical variables was the first category, their values were not calculated, Bold marked p-values are used to highlight statistical significance

With the logistic regression analysis, esports players’ risk factors for developing food addiction were evaluated (Table 5). These results indicate that perceived poor sleep quality may be associated with a 2.12 times increase in food addiction (OR: 2.121, *p* = 0.058). The analysis of the factors that increased the risk of food addiction in esports players demonstrated that an hourly increase in weekly esports playing time increased the risk of food addiction 1.02 times (OR:1.022, *p* < 0.05). The analysis also demonstrated that the risk of becoming addicted to food was 0.4 times higher in smoker esports players than it was in non-smokers (OR: 0.405, *p* < 0.05)

**Table 5:** Univariate binary logistic regression analysis for food addiction versus no food addiction

Variable	Coef (B)	S.E.	Odds Ratio (Exp (B))	95 CI Upper Lower	p-value
**Esports level** Amateur esports players* Professional esports players	0.560	0.378	1.751	0.835–3.675	0.138
**Weekly esports participation time (hours/week)**	0.021	0.010	1.022	1.001–1.043	**0.041**
**Smoking status** Non-smoker * Smoker	0.904	0.377	0.405	0.193-0.848	**0.017**
**Alcohol consumption** No* Yes	0.416	0.379	1.515	0.721–3.185	0.273
**Physical Activity**	0.467	0.438	0.627	0.677–3.760	0.286
**The number of main meals** 1* 2 + 3	-0.413 -0.602	0.614 0.383	0.662 0.547	0.199–2.203 0.258–1.160	0.501 0.116
**Skipping meals** No* Yes	-0.501	0.403	0.606	0.275–1.336	0.214
**Perceived sleep quality** Good* Bad	0.752	0.397	2.121	0.974–4.618	0.058
**BMI** Underweight (< 18.5)* Normal (18.5–24.9) Overweight (25–30) People with obesity (≥ 30)	-1.331 -0.475 -0.145	0.934 0.496 0.554	0.264 0.622 0.865	0.042–1.647 0.235–1.643 0.292–2.564	0.154 0.338 0.794

Logistic regression analysis, OR: odds ratio; CI: confidence interval

*Because the “reference” category for the categorical variables was the first category, their values were not calculated, Bold marked p-values are used to highlight statistical significance

## Discussion

While interest in esports continues to surge worldwide, research on the associated health problems remains scarce [26–28]. Malnutrition, mental fatigue, psychological issues, sedentary lifestyles, and increased BMI are among the prevalent health concerns for esports players [29]. Our study aimed to pioneer the investigation of esports players’ daily living conditions, focusing on two eating disorders: Night Eating Syndrome (NES) and food addiction. A systematic review on esports players’ health identified a sedentary lifestyle as the primary risk factor [12]. Surprisingly, our study found that 77% of participating esports players engaged in moderate physical activity, with an average of 78 h of inactivity per week (including sleep). This suggests that players attempt to compensate for prolonged sitting with moderate activity. In contrast, previous studies reported that esports players remained inactive during training for an average of 4.2 h per day [30]. Additionally, professional esports players were found to engage in approximately 1 h of physical activity per day, exceeding WHO guidelines [30–32]. Overall, our study contributes to the understanding of esports players’ health by shedding light on their daily activity levels and addressing prevalent eating disorders. Further research is needed to explore interventions aimed at promoting healthier lifestyles within the esports community

In low- and middle-income countries, the incidence of game addiction among esports players is relatively low. However, despite this, they often encounter stress, sleep-related issues, and psychological conditions related to self-perception [33]. Prolonged screen exposure raises concerns about sleep disorders and disrupts the circadian rhythm in esports players [34]. Notably, esports players tend to have shorter sleep durations compared to healthy adults [35]. In our study, participants reported an average sleep duration of 52 h per week, with 70% reporting good sleep quality

Studies on esports suggest that players spend approximately 5–6 h per day gaming [30, 36]. A multi-center study including players from Turkey, South Korea, and the USA reported a mean BMI of 26.03 ± 1.85 kg/m2 and a daily gaming duration of 9.34 ± 1.12 h [37]. Moreover, higher gaming durations have been associated with increased BMI [37]. However, in our study, the mean BMI and weekly gaming time were lower than those reported in the literature. This could be attributed to our participants being university students, who may have limited time for gaming due to academic commitments and may also lead more physically active lifestyles

Playing video games is associated with skipping meals, particularly among men. A study reported that individuals who play video games at least four times a week are nine times more likely to skip meals than those who never play [38, 39]. There is a positive association between playing esports for long hours, and malnutrition and night eating behaviors [40]. As was demonstrated in a previous study on eating habits, increased frequency of nighttime eating was associated with decreased morning hunger and increased breakfast skipping [41]. Similarly, in our study, the meal skipped most was breakfast (41.9%). According to the results of our logistic regression analysis, skipping meals is a factor that is associated with higher the frequency of NES in esports players

In our study, rates of smoking and alcohol use in the participating esports players were 37.0% and 41.0%, respectively. Although there is no population data on esports players’ smoking and alcohol use habits, a linear relationship has been shown between average video game playing time and alcohol, nicotine, and substance use in adults [42]. A study with esports players found a smoking frequency of 8% [43]. Smoking esports players had a 0.4 times higher risk of food addiction compared to non-smokers. Each 1-hour increase in weekly esports gaming time was associated with a 1.02 times higher risk of food addiction. Compared to those with good perceived sleep quality, the risk of food addiction was 2.2 times higher in those with poor perceived sleep quality. Therefore, it is important to conduct more studies in which food addiction is clinically assessed and the relationship between food addiction and risk factors are confirmed if a sports person’s health is to be improved

It is clear that prolonged gaming is linked to poor eating habits [44]. A study comparing dietary habits between esports players and non-players found that esports players expressed less concern about following a balanced diet and consumed fewer portions of fruits and vegetables than non-players [45]. A study conducted by Szot et al. (2022) revealed that esports players exhibited poor eating habits, including irregular meal times, frequent snacking, frying of meat dishes, and sweetening of hot drinks. It was also found that the consumption of products with potential beneficial effects on brain function such as fish, nuts, pumpkin seeds, sunflower seeds and other oilseeds was low [46]. Additionally, another study found that esports players had low adherence to the Mediterranean dietary pattern, consumed fewer fruits and vegetables, and had high consumption of fast food, red and processed meat, and soft drinks [47]. Unhealthy eating habits in esports players may predispose to obesity, heart disease or metabolic disorders [48]. On the other hand, considering that esports is generally more common among young people, inappropriate eating habits that persist throughout middle and late adulthood may lead to negative health consequences that are irreversible or difficult to reverse [47]. In this study, the fact that most of the esports players skipped meals, 38.4% had a BMI of 25 and above, and a considerable level of food addiction and NES showed that they had unhealthy eating habits. These findings suggest that esports players are vulnerable to eating disorders and many metabolic diseases, including obesity

Because our study is one of the pioneering studies in which the relationship between NES and food addiction in esports players is investigated, we compared our results with those of studies in which these addictions were investigated in young adults. There is evidence that the frequency of NES is around 4–5% in young university students aged 18–25 years [49–51]. In a recent study conducted in Palestine, the frequency of NES among students was reported as 29.7%. NES positivity was statistically significantly high in men (compared to women), those with mental health problems, and those with financial problems. That the frequency of NES in the aforementioned study was higher than it was in other studies in the literature was probably due to the changing living conditions and the time spent watching sports matches after the pandemic [52]. Our study revealed a NES prevalence of 13.3%. One possible explanation for why our study yielded this result could be the unique nature of the esports environment, which may entail distinct lifestyle factors and stressors compared to traditional young adult populations surveyed in previous studies

Studies on NES have provided evidence suggesting the involvement of potential genetic risk factors in the etiology of the disease, with a relatively higher susceptibility observed in men, possibly due to the predominance of men among esports players [53–55]. It is assumed that individuals with NES consume about a quarter of their daily energy intake at night. In experimental studies, food craving and binge-eating responses of mice exposed to a high-fat and high-sugar diet to sleep delay were observed, and it was demonstrated that the response to food addiction could be measured neurally in those exposed to sleep delay [56, 57]. Our study revealed that half of the esports players with night eating syndrome also exhibited a statistically significant level of food addiction. The association between NES and YFAS was first reported by Nolan & Geliebter [17] and confirmed by Taymur et al. [58]. The prevalence of food addiction and night eating syndrome in the young Turkish population without obesity was found to be 2%, and a positive correlation was found between YFAS and NEQ [58]. We predict that the relationship between night eating and food addiction in esports players may be at a higher level than it is in other populations. In a study on esports including participants the majority were under 30 years old, men, and often underweight or had normal body weight, no statistically significant relationship was determined between nomophobia, physical activity, and food addiction. However, there was a positive relationship between nomophobia symptoms, and insomnia and stress factors [15]. Esports players may be considered a group at risk for NES, especially considering their exposure to factors such as sleep disturbances and stress. In our study, it was determined that associated with night eating was observed as the increase in weekly duration of playing esports and skipping meals

We, the authors, acknowledge that the present study has some limitations. First, causality was not determined due to the descriptive correlational design of the study. Another one is that the data was collected based on self-report. As is known, individuals can report their body weight and height values differently than they are [59]. The YFAS, on the other hand, can only provide symptoms, but clinical diagnosis can be made through consultations with a healthcare professional. The fact that our country-specific randomization was not defined in the selection of the sample also causes low representativeness and makes interpretation and generalizability controversial in reflecting our country’s esport profile. However, our findings can be considered as an additional source for literature on esports players’ food addiction and NES status. The study can also lay the groundwork for planning intervention programs and even case-control studies for the identification and monitoring of food addiction by the health professional

## Conclusion

In our study, we determined that skipping meals is associated with higher night eating syndrome in esports players, that perceived poor sleep quality and smoking is associated with higher food addiction, and that weekly duration of playing esports is associated with higher both night eating syndrome and food addiction. We observed that of the esports players, those with NES were at risk of food addiction, and that night eating syndrome was quite common in esports players. Given the different features of esports, which are developing and becoming more and more popular than traditional sports, we suggest that esports players should be evaluated in terms of their unhealthy eating behaviors and risk of eating disorders. We also advocate for the implementation of initiatives to promote healthy eating habits of esports players
